# Reusable Surface-Enhanced Raman Spectroscopy Substrates Made of Silicon Nanowire Array Coated with Silver Nanoparticles Fabricated by Metal-Assisted Chemical Etching and Photonic Reduction

**DOI:** 10.3390/nano9111531

**Published:** 2019-10-28

**Authors:** Shi Bai, Yongjun Du, Chunyan Wang, Jian Wu, Koji Sugioka

**Affiliations:** 1 Institute of Laser Engineering, Beijing University of Technology, 100 Pingle Yuan, Beijing 100124, China; shibaibjut@gmail.com (S.B.); duyongjun@emails.bjut.edu.cn (Y.D.); 2 Advanced Laser Processing Research Team, RIKEN Center for Advanced Photonics, RIKEN, 2–1 Hirosawa, Wako, Saitama 351–0198, Japan; 3 State Key Laboratory of Space Medicine Fundamentals and Application, Chinese Astronaut Science Researching and Training Center, Beijing, 100094, China; cywang16@sina.com

**Keywords:** nanostructure, reusability, photonic reduction, SERS, metal assisted chemical etching

## Abstract

Surface-enhanced Raman spectroscopy (SERS) has advanced over the last four decades and has become an attractive tool for highly sensitive analysis in fields such as medicine and environmental monitoring. Recently, there has been an urgent demand for reusable and long-lived SERS substrates as a means of reducing the costs associated with this technique To this end, we fabricated a SERS substrate comprising a silicon nanowire array coated with silver nanoparticles, using metal-assisted chemical etching followed by photonic reduction. The morphology and growth mechanism of the SERS substrate were carefully examined and the performance of the fabricated SERS substrate was tested using rhodamine 6G and dopamine hydrochloride. The data show that this new substrate provides an enhancement factor of nearly 1 × 10^8^. This work demonstrates that a silicon nanowire array coated with silver nanoparticles is sensitive and sufficiently robust to allow repeated reuse. These results suggest that this newly developed technique could allow SERS to be used in many commercial applications.

## 1. Introduction

The three-dimensional (3D) surface-enhanced Raman spectroscopy (SERS) substrate is a powerful analytical method with applications in the fields of medicine, environmental monitoring, and spectral imaging [1,2,3,4]. Compared to two-dimensional (2D) SERS substrates, the 3D SERS substrate offers two unique advantages [5,6]. Specifically, because a 3D SERS substrate provides a larger surface area and a higher density of so-called “hot spot” zones along the vertical direction compared to a flat 2D substrate, the former allows increased sensitivity and greater enhancement. The other advantage is that a 3D SERS substrate has a more wettable nanostructure, which will improve the SERS sensitivity and repeatability [7,8]. A 3D SERS substrate is typically composed of a combination of nanowires and metallic nanoparticles. The metallic nanoparticles act as sensing units to generate hot spots in association with localized surface plasmon resonance, which greatly enhances the extent of Raman scattering [9]. The nanowires serve as the backbone of the 3D structure and the nanowire array also serves to further increase the repeatability of the Raman signal due to its periodic structure [10]. As an example, Oh et al. reported a 3D SERS substrate comprising a glass nanopillar array with silver nanoislands [11]. This substrate was found to have a high enhancement factor (EF) and increased the SERS signals by more than one order of magnitude compared to a 2D SERS substrate. This improved performance was attributed to the ability of the silver nanoislands to generate a high density of hot spots on both the top and side of the glass nanopillar array [11]. Park et al. used an Ar^+^ plasma to treat a polyethylene terephthalate surface to form polymeric nanopillars. Gold nanoparticles were subsequently generated by evaporation to create a 3D gold-rich nanopillar structure that induced the self-clustering of plasmonic nanogaps due to capillary forces and increased the concentration of analyte on the substrate surface [12]. In 2018, Wang et al. developed a 3D gold-rich nanopillar plasmonic substrate for use in SERS analyses that anchored mycotoxins via nanotags. Their work determined that the surface tension induced a leaning effect in groups of two or three adjacent nanopillars that resulted in the self-assembly of hot spots, which, in turn, significantly enhanced the SERS signal [13].

Some SERS substrates, typically containing materials such as TiO_2_ and ZnO, can be reused following a self-cleaning process based on the photocatalytic degradation of residual analyte. Following the SERS analysis, such substrates are exposed to either ultraviolet (UV) or visible (VIS) light to degrade residual analyte molecules, after which ions are removed using water wash. Based on this procedure, the refreshed SERS substrate can be reused several times. However, the structure and morphology of a metal layer deposited on a 3D structure are difficult to control, which results in a relatively low SERS enhancement.

In the present work, we developed a simple means of fabricating the 3D SERS substrates, based on coating silicon nanowire arrays with silver nanoparticles (Ag-NP/Si-NW). The morphology of these SERS substrates, which is critical to their performance, could be controlled using metal-assisted chemical etching (MACE) followed by photonic reduction. Herein, we discuss the formation of silver nanoparticles via photonic reduction and their contribution to the SERS enhancement. These 3D SERS substrates are also shown to be reusable after employing UV radiation to degrade dye molecules following an analysis. Finally, these 3D SERS substrates were applied for the analysis of dopamine (DA) to verify that they provide both high sensitivity and reliability.

## 2. Experimental Section

### 2.1. Silicon Nanowire Array Fabrication by Metal-Assisted Chemical Etching

As shown in Supporting Information, Figure S1, n-type silicon wafers (resistivity of 1–10 Ω·cm) were ultrasonically cleaned using deionized water and ethanol. The cleaned silicon wafers were then immersed in a 5% HF solution to remove oxide layers and generate H-terminated surfaces. The wafers were immediately transferred to a solution containing 4.8 M HF and 0.005 M AgNO_3_ and held motionless for 1.5 min. After the wafer surfaces transitioned to an off-white coloration (meaning that silver nanoparticles covered the wafer surfaces at a high density), they were immersed in etching solution containing 4.8 M HF and 0.1 M H_2_O_2_. The MACE process was carried out for 30 min at room temperature to produce silicon nanowires. After this etching step, the wafers were immediately immersed in a 30 wt.% HNO_3_ solution to dissolve the Ag nanoparticles, then rinsed with deionized water.

### 2.2. Silver Nanoparticles Synthesized by Photonic Reduction

In a typical synthesis, 425 mg AgNO_3_ (equivalent to 10 mM), 36.8 mg sodium citrate (equivalent to 0.5 mM), and 0.5 ml of a 40% HF solution were dissolved in 250 ml deionized water. The silicon nanowire arrays formed using the procedure described in Section 2.1 were then placed into an open vessel together with 15 ml of this mixture and irradiated with a UV lamp (365 nm), resulting in silicon nanowire surfaces coated with silver nanoparticles. The power density of the UV lamp was set at 46 mW/cm^2^.

### 2.3. SERS Analyses

The SERS performance obtained using these Ag-NP/Si-NW arrays was assessed in conjunction with a Raman spectrometer (in Via-Reflex, Renishaw, United Kingdom) equipped with a 50× objective lens. The wavelength of the excitation laser was 532 nm and the laser power and integration time were set at 0.5 mW and 10 s, respectively. The SERS substrate was first placed in 5 mL of 10^−4^ M rhodamine 6G aqueous solution, then irradiated with UV light for 120 min while stirring with a magnetic stirrer. Finally, the SERS substrate was taken out and rinsed with deionized water and air dried, and SERS spectra were recorded for the substrate in the dry state.

### 2.4. Characterization

The structures and morphologies of the substrates were investigated by scanning electron microscopy (SEM, Hitachi S-4800, Japan) and transmission electron microscopy (TEM, Tecnai G2 F20, FEI, US), respectively. X-ray diffraction (XRD) patterns for the prepared substrates were also acquired at room temperature (D8 Advance, German), and the absorption spectra were obtained using a UV-Vis spectrophotometer (Metash UV-9000S, Shanghai, China).

## 3. Results and Discussion

**Figure 1:**
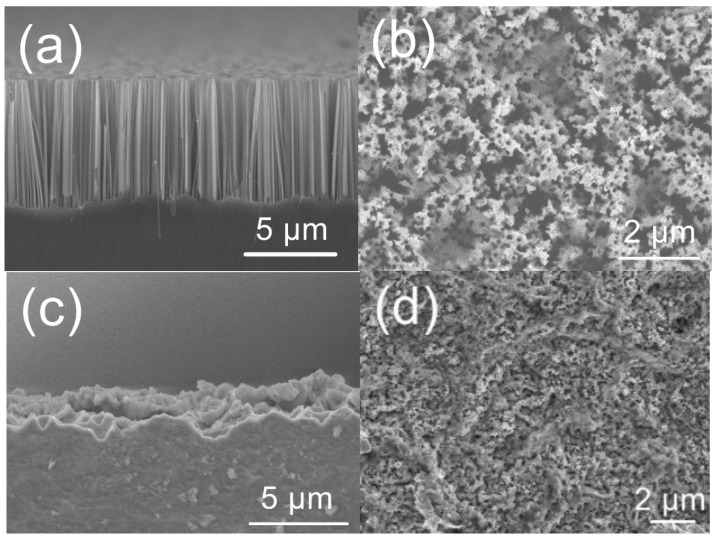
(**a**,**c**) Cross-sectional and (**b**,**d**) top SEM images of silicon morphology following metal-assisted chemical etching (MACE) procedure. (**a**,**b**) Silicon nanowire array etched with 4.8 M HF and 0.1 M H_2_O_2_. (**c**,**d**) Irregular silicon structure etched with 4.8 M HF and 10 M H_2_O_2_.

Figure 1a,b present cross-sectional and top-view SEM images of a silicon nanowire array formed by the MACE process. The mean length of the silicon nanowires was approximately 10 μm and the gap between each nanowire was approximately 400 nm. The overall reaction associated with the MACE process is well known and can be summarized as [14]

(1) $$\mathrm{Si}+2H_{2}O_{2}+6\mathrm{HF}\to H_{2}\mathrm{Si}F_{6}+4H_{2}O.$$

The silver nanoparticles deposited on the silicon wafer prior to chemical etching attract electrons from the silicon and thus promote the oxidation of the wafer to SiO_2_. This SiO_2_ is subsequently removed by etching with the HF to generate nanostructures on the wafer surface. This etching of the silicon is primarily the result of two reactions. The first reaction can be summarized as [15]

(2) $$\mathrm{Si}+4{\mathrm{Ag}}^{+}+6F^{-}\to 4\mathrm{Ag}+\mathrm{Si}F_{6}^{2-},$$

while the other is [15,16]

(3) $$2\mathrm{Ag}+H_{2}O_{2}+2H^{+}\to 2{\mathrm{Ag}}^{+}+2H_{2}O.$$

From Equations (2) and (3), it is evident that the HF:H_2_O_2_ ratio in the etching solution will affect the resulting morphology. According to the reaction of MACE, the Ag nanoparticles work as the catalyst. Then, the etching process is mainly determined by the volumes of HF and H_2_O_2_ [14,17,18]. The silicon is oxidized by HF at the side of the Ag nanoparticles facing the silicon, and the silicon oxide is dissolved by HF in the etching solution. This oxidation and dissolution make the etching process sustain and generate Si nanowires. As an example, in the case of an etching solution with a moderate ratio (Figure 1a,b), the silicon surface will be oxidized and then etched by the HF with the assistance of the Ag nanoparticles. The oxidation/etching reaction proceeds in a continuous process, resulting in the silicon under the Ag nanoparticles gradually becoming etched to a greater depth such that silicon nanowires are eventually formed. However, at a higher HF:H_2_O_2_ ratio, reaction (2) will become dominant over reaction (3), such that silver atoms will be generated faster than these atoms can dissolve until the H_2_O_2_ is completely consumed. The silver atoms produced in this manner will form new silver nanoparticles that trigger the etching of the silicon sidewalls, such that the etching direction is no longer controlled. This uncontrollable etching process induces both vertical and lateral etching, resulting in an irregular silicon structure (Figure 1c,d) [19].

**Figure 2:**
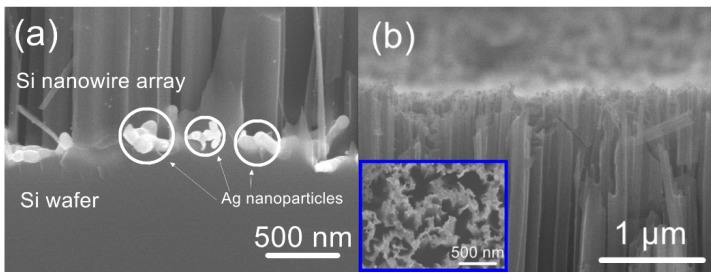

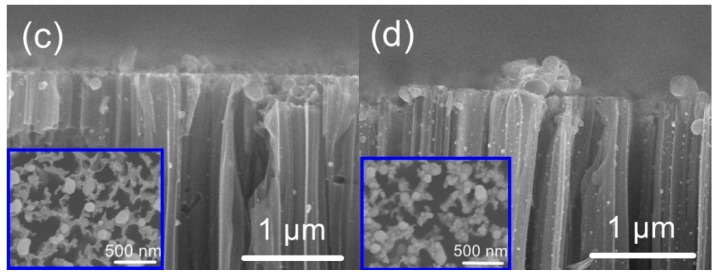
(**a**) SEM images of silicon nanowires covered with Ag particles at bottom of silicon nanowire array. (**b**–**d**) SEM images of silicon nanowire array coated with silver nanoparticles following UV irradiation for 2, 5, and 10 min, respectively. The concentration of Ag^+^ in the solution was 2.5 mM.

Before photonic reduction, the Ag^+^ precursor must permeate to the bottom of the silicon nanowire. Therefore, a trace of HF was added in the precursor solution to ensure that the silver nanoparticles would form over the entirety of the sidewalls of the silicon nanowires during UV irradiation. Figure 2a demonstrates that silver particles were indeed formed at the bottom of the nanowires and were, in fact, much larger than those formed on the sidewalls. This effect can be explained by considering that the concentration of HF in the Ag^+^ precursor solution was insufficient to promote continuous silicon etching. The HF preferentially reacted with silicon and Ag^+^ to form Ag particles on the silicon wafer surfaces having a (100) orientation because of the higher probability of electrons being captured by metal ions on these surfaces [15,19,20]. Figure 2b–d present the morphologies of the Ag-NP/Si-NW arrays obtained following different UV irradiation times. With increasing irradiation time, more silver nanoparticles were found to cover the silicon nanowires, and the mean size of these nanoparticles also increased. This occurred because the Ag^+^ ions attracted electrons from citrate and were reduced to Ag(0) in response to the UV irradiation [21]. Eventually, the Ag nanoparticles on the tips of the silicon nanowire array became four times larger than those on the silicon nanowire sidewalls (Figure 2d and Supporting Information, Figure S2). This size difference is ascribed to the limited space in the gaps between the nanowires that restricted the growth of silver nanoparticles, as well as the blocking of incident photons by the Ag nanoparticles formed on the tips. As a result of this blocking effect, the generation of silver atoms in the gaps between the nanowires occurred primarily as a result of scattered UV light [22].

**Figure 3:**
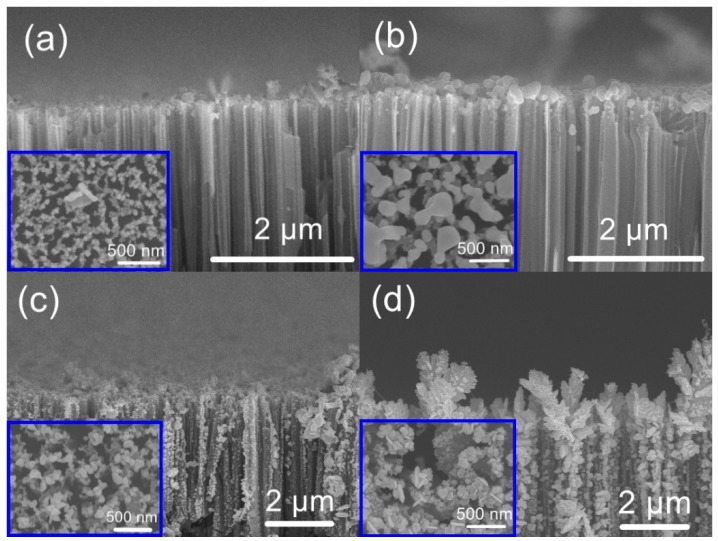
SEM images of silicon nanowire arrays coated with silver nanoparticles using Ag^+^ solutions with concentrations of (**a**) 1.25, (**b**) 2.5, (**c**) 5, and (**d**) 10 mM in conjunction with a UV irradiation time of 10 min.

Figure 3 demonstrates the photonic reduction of silver nanoparticles for various Ag^+^ concentrations under UV irradiation. The nanoparticle size evidently increased as the concentration of Ag^+^ was increased. The presence of citrate ions limited the Ag nanoparticle growth at lower Ag^+^ concentrations, while larger particles were obtained at higher Ag^+^ concentrations, as a result of the aggregation of smaller silver nanoparticles [2,23]. At an Ag^+^ concentration of 10 mM, the silver particles grew away from the silicon nanowire array surface to form branched silver dendrites that were vertically embedded in the array. These dendrites were due to the aggregation of small groups of silver nanoparticles in self-supporting configurations. Notably, these branched silver dendrites contained nanogaps and neck-like areas due to the random aggregation of silver nanoparticles [2,24].

**Figure 4:**
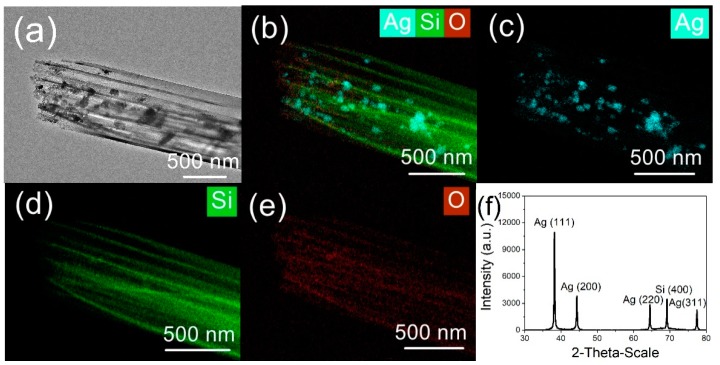
(**a**) TEM image of silicon nanowire array coated with silver nanoparticles. (**b**–**e**) EDS mapping images of this array: (**b**) combined Ag, Si, and O map; (**c**) Ag map; (**d**) Si map; (**e**) O map. (f) XRD pattern obtained from this array. The Ag^+^ concentration used was 1.25 mM and the UV irradiation time was 10 min.

Figure 4 shows TEM images and an energy-dispersive X-ray spectroscopy (EDS) map of the Ag-NP/Si-NW array produced using the MACE with subsequent photonic reduction. The TEM image clearly shows silver nanoparticles attached to the tips and sidewalls of the silicon nanowires. Figure 4b–e present EDS maps for Si, Ag, and O. These data confirm that silicon was situated in the middle of the structure as the backbone, with silver surrounding the silicon nanowires to form the Ag-NP/Si-NW. The distribution of silver on the silicon nanowires is not homogenous, due to the low concentration of the silver nanoparticles. It should also be noted that, because the Si nanowire array was not treated with HF after the MACE process to remove the oxidation layer, O was also detected by EDS on the outer layers of the nanowires. In prior work, this oxidation layer has been shown to affect the photoluminescence properties of the material [17,25,26]. XRD analysis of an Ag-NP/Si-NW array produced sharp peaks that can be assigned to diffraction from the (111), (200), (220), and (311) planes of face-centered cubic (FCC) silver [27]. Additionally, the broad peak centered at 69° resulting from silicon and slightly shifted from 70° is associated with diffraction from (400) planes, which indicates that the silicon was in the form of a nanowire array [28].

**Figure 5:**
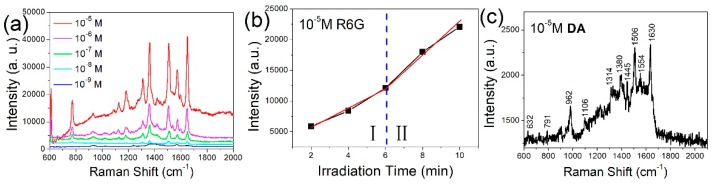
(**a**) Surface-enhanced Raman spectroscopy (SERS) spectra of Rhodamine (R6G) solutions with different concentrations on silicon nanowires coated with silver nanoparticles. (**b**) Raman intensity at 612 cm^−1^ for 10^−5^ M R6G as a function of UV irradiation time. (**c**) Raman spectrum of 10^−5^ M dopamine hydrochloride solution. The laser power for dopamine (DA) Raman measurement was 0.01 mW.

The performance of these Ag-NP/Si-NW array SERS substrates was examined using Rhodamine (R6G) solutions and DA as model compounds. Figure 5a presents the Raman spectra obtained from R6G solutions having concentrations from 10^−5^ to 10^−9^ M on an Ag-NP/Si-NW array fabricated using the MACE process followed by photonic reduction with UV irradiation for 10 min. The Raman intensity for the R6G solution naturally decreased with decreasing concentration, and Raman peaks could no longer be identified at 10^−9^ M R6G. To quantitatively evaluate the performance of the SERS substrate, the analytical EF value was calculated using [29]

(4) $$\mathrm{EF}=\left(I_{\mathrm{SERS}}/I_{\mathrm{OR}}\right)/\left(C_{\mathrm{SERS}}/C_{\mathrm{OR}}\right)$$

where I_SERS_ and I_OR_ correspond to the Raman intensities for R6G on an Ag-NP/Si-NW array SERS substrate and a silicon wafer at the detection limits, respectively, and C_SERS_ and C_OR_ are the R6G concentrations at the detection limits on the Ag-NP/Si-NW array SERS substrate and silicon wafer, respectively. The Raman spectrum produced by R6G on a silicon wafer is provided in Supporting Information, Figure S3. It is notable that under 532 nm laser irradiation, R6G would be resonantly excited as R6G has a strong absorption near this wavelength [30]. However, the resonant Raman intensity is proportional to the number of R6G molecules [31]. Compared to SERS, the resonant Raman intensity is quite weak when the concentration of R6G is about 10^−9^ M. Therefore, we ignored the resonant Raman effect when we calculated the SERS enhancement factor. In addition, because the SERS substrate we prepared was a three-dimensional substrate (the number of excited molecules in the vertical direction is difficult to estimate, because the number cannot be directly deduced from experimental results), the SERS EF of the substrate was calculated by the detection limit of concentration instead of molecule numbers. According to Equation (4), the SERS EF associated with the Ag-NP/Si-NW array was determined to be as high as 1.4 × 10^8^. In addition, we investigated variations in the Raman intensity obtained from R6G on the Ag-NP/Si-NW array fabricated using different UV irradiation times, as shown in Figure 5b. The Raman intensity increased with UV irradiation time because more silver nanoparticles were generated on the silicon nanowires, which, in turn, increased the total quantity of excited R6G molecules. Notably, the increase in Raman intensity was especially enhanced after 6 min UV irradiation (Region II in Figure 5b). This acceleration of the Raman intensity is attributed to hot spots generated in the spaces between adjacent silver nanoparticles on the branched silver dendrites. These spots effectively enhance the local electric field and boost the emission from analyte molecules in the nanogap and neck areas. This effect has also been demonstrated in previous studies (Supporting Information, Figure S4) [2,11,32,33]. Finally, we used the Ag-NP/Si-NW as a SERS substrate for the detection of the neurotransmitter DA. Figure 5c shows the Raman spectrum obtained from a 10^−5^ M DA solution using our prepared SERS substrate, and the detection limit is lower than 10^−7^ M DA solution (Supporting information, Figure S5). The main Raman peaks of DA were successfully identified, including those at 1106, 1554, and 1630 cm^−1^ ascribed to the in-plane –OH bending vibration, and the in-plane bending vibration arising from the benzene ring and hydroxyl, respectively [34,35].

Reusability of the SERS substrate has become of interest in recent years, as recycling SERS substrates reduces costs. After a measurement is performed, the SERS substrate can be treated using several methods, including heating or acid cleaning, to eliminate the analyte in preparation for the next measurement [36,37]. In prior work, dye molecules on a silicon nanowire array were decomposed by photocatalytic degradation with the assistance of metal nanoparticles, to permit the self-cleaning of a SERS substrate [38]. To illustrate the reusability of the present Ag-NP/Si-NW array, we investigated reuse via the photocatalytic degradation for R6G molecules.

**Figure 6:**
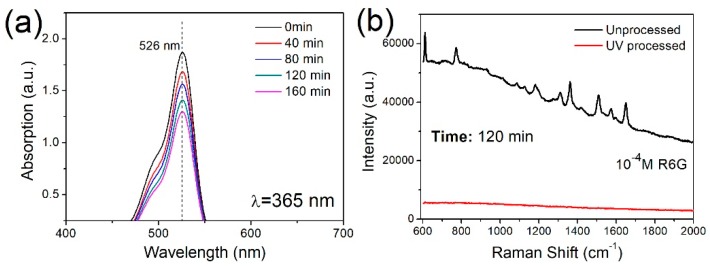
(**a**) UV-Vis absorption spectra of R6G solutions following UV irradiation with different times on the surface of Ag nanoparticle-coated silicon nanowire array. (**b**) Raman spectra of 10^−4^ M R6G solution before (black) and after (red) 120 min UV photocatalytic degradation.

Figure 6a shows the UV-vis absorption spectra of an R6G solution during photocatalytic degradation over various time spans using the Ag-NP/Si-NW array. The maximum absorbance of R6G is located at 526 nm, and this peak decreased in intensity as the UV irradiation time was prolonged. In a metal–semiconductor catalysis system, photocatalytic degradation primarily depends on the electron transfer process. Due to the wide energy band and high recombination rate of electrons and holes in the silicon nanowires under UV irradiation, the silver nanoparticles were used as an accelerant to enhance the photocatalytic activity. Specifically, under UV irradiation, both electrons and holes were generated in the silicon nanowires, after which the electrons were transferred to these nanoparticles because the Fermi level of these nanoparticles (–1.8 V) was below the conduction band of the silicon nanowires (–0.69 V) [39,40]. The photogenerated electrons reacted with O_2_ absorbed on the Ag nanoparticles to form superoxide radical ions $O_{2}^{•-}$, and subsequently produced $HO_{2}^{•}$ and $OH_{}^{•}$ radicals. In addition, holes in the valence band of the silicon nanowires received electrons from water and generated $HO_{}^{•}$ radicals. These oxidants caused rapid degradation of the R6G molecules without damaging the structure of the Ag-NP/Si-NW array (Supporting Information, Figure S6) [28,39].

To illustrate the reusability of the Ag-NP/Si-NW array by self-cleaning, the used SERS substrate was exposed to UV irradiation for 120 min and then again applied to the analysis of R6G. Figure 6b demonstrates the disappearance of the characteristic R6G after the UV light cleaning, indicating that the R6G molecules were decomposed by active oxidants on the Ag-NP/Si-NW array. For comparison purposes, a used SERS substrate was instead immersed in deionized water in a dark room for 120 min. Raman peaks due to R6G were subsequently identified, confirming that the R6G molecules had not been removed. The reusability of our SERS substrate was also assessed using DA. Similar results confirmed the removal of the DA, with much less intense Raman peaks after 120 min UV photodegradation compared to 60 min UV photodegradation (Supporting Information, Figure S7). Thus, we can conclude that the R6G and DA molecules were detached from the Ag-NP/Si-NW array by photocatalytic degradation under UV irradiation, enabling us to reuse the array for repeated SERS measurements.

## 4. Conclusions

Reusable 3D SERS substrates based on Ag-NP/Si-NW arrays were fabricated and found to allow the analysis of DA at low concentrations. The silicon nanowire arrays in these substrates were fabricated using the MACE process, and subsequently coated with Ag nanoparticles by photonic reduction. Because these substrates generated hot spots along the sidewalls of the silicon nanowires, the EF associated with SERS analyses used was as high as 1 × 10^8^. These substrates could also be reused following self-cleaning under UV radiation. This novel 3D SERS substrate concept could potentially allow the fabrication of low-cost, sensitive, and reusable SERS sensors.

## Supplementary Materials

The supplementary materials are available online at https://www.mdpi.com/2079-4991/9/11/1531/s1.

## References

[1] Zengin A., Tamer U., Caykara T. (2013). A SERS-Based Sandwich Assay for Ultrasensitive and Selective Detection of Alzheimer’s Tau Protein. Biomacromolecules.

[2] Bai S., Lin Y.H., Zhang X.P., Zhou W.P., Chen T., Ma Y., Hou T.X., Bridges D., Oakes K., Hu A.M. (2015). Two-Step Photonic Reduction of Controlled Periodic Silver Nanostructures for Surface-Enhanced Raman Spectroscopy. Plasmonics.

[3] Strozyk M.S., de Aberasturi D.J., Gregory J.V., Brust M., Lahann J., Liz-Marzan L.M. (2017). Spatial Analysis of Metal-PLGA Hybrid Microstructures Using 3D SERS Imaging. Adv. Funct. Mater..

[4] Bai S., Serien D., Hu A.M., Sugioka K. (2018). 3D Microfluidic Surface-Enhanced Raman Spectroscopy (SERS) Chips Fabricated by All-Femtosecond-Laser-Processing for Real-Time Sensing of Toxic Substances. Adv. Funct. Mater..

[5] Huang J.A., Zhao Y.Q., Zhang X.J., He L.F., Wong T.L., Chui Y.S., Zhang W.J., Lee S.T. (2013). Ordered Ag/Si Nanowires Array: Wide-Range Surface-Enhanced Raman Spectroscopy for Reproducible Biomolecule Detection. Nano Lett..

[6] Ling D.D., Wu Z.L., Li S.J., Zhao W.Q., Ma C.J., Wang J., Jiang Z.M., Zhong Z.Y., Zheng Y.B., Yang X.J. (2017). Large-Area Au-Nanoparticle-Functionalized Si Nanorod Arrays for Spatially Uniform Surface-Enhanced Raman Spectroscopy. ACS Nano.

[7] Wang W.D., Yin Y.G., Tan Z.Q., Liu J.F. (2014). Coffee-ring effect-based simultaneous SERS substrate fabrication and analyte enrichment for trace analysis. Nanoscale.

[8] Zhao H.Y., Jin J., Tian W.J., Li R., Yu Z., Song W., Cong Q., Zhao B., Ozaki Y. (2015). Three-dimensional superhydrophobic surface-enhanced Raman spectroscopy substrate for sensitive detection of pollutants in real environments. J. Mater. Chem. A.

[9] Liu J., Meng G.W., Li Z.B., Huang Z.L., Li X.D. (2015). Ag-NP@Ge-nanotaper/Si-micropillar ordered arrays as ultrasensitive and uniform surface enhanced Raman scattering substrates. Nanoscale.

[10] Tan E.Z., Yin P.G., You T.T., Wang H., Guo L. (2012). Three Dimensional Design of Large-Scale TiO_2_ Nanorods Scaffold Decorated by Silver Nanoparticles as SERS Sensor for Ultrasensitive Malachite Green Detection. ACS Appl. Mater. Interfaces.

[11] Oh Y.J., Jeong K.H. (2012). Glass Nanopillar Arrays with Nanogap-Rich Silver Nanoislands for Highly Intense Surface Enhanced Raman Scattering. Adv. Mater..

[12] Park S.G., Mun C., Xiao X.F., Braun A., Kim S., Giannini V., Maier S.A., Kim D.H. (2017). Surface Energy-Controlled SERS Substrates for Molecular Concentration at Plasmonic Nanogaps. Adv. Funct. Mater..

[13] Wang X.K., Park S.G., Ko J., Xiao X.F., Giannini V., Maier S.A., Kim D.H., Choo J. (2018). Sensitive and Reproducible Immunoassay of Multiple Mycotoxins Using Surface-Enhanced Raman Scattering Mapping on 3D Plasmonic Nanopillar Arrays. Small.

[14] Lu Y.T., Barron A.R. (2013). Nanopore-type black silicon anti-reflection layers fabricated by a one-step silver-assisted chemical etching. Phys. Chem. Chem. Phys..

[15] Zhang M.L., Peng K.Q., Fan X., Jie J.S., Zhang R.Q., Lee S.T., Wong N.B. (2008). Preparation of large-area uniform silicon nanowires arrays through metal-assisted chemical etching. J. Phys. Chem. C.

[16] Li S.Y., Ma W.H., Zhou Y., Chen X.H., Xiao Y.Y., Ma M.Y., Zhu W.J., Wei F. (2014). Fabrication of porous silicon nanowires by MACE method in HF/H_2_O_2_/AgNO_3_ system at room temperature. Nanoscale Res. Lett..

[17] Wu J., Du Y., Wang C., Bai S., Zhang T., Chen T., Hu A. (2019). Reusable and long-life 3D Ag nanoparticles coated Si nanowires array as sensitive SERS substrate. Appl. Surf. Sci..

[18] Chen C., Wong C. (2015). Unveiling the shape-diversified silicon nanowires made by HF/HNO 3 isotropic etching with the assistance of silver. Nanoscale.

[19] Srivastava S.K., Kumar D., Schmitt S.W., Sood K.N., Christiansen S.H., Singh P.K. (2014). Large area fabrication of vertical silicon nanowire arrays by silver-assisted single-step chemical etching and their formation kinetics. Nanotechnology.

[20] Wang Y., Yang L., Liu Y., Mei Z., Chen W., Li J., Liang H., Kuznetsov A., Du X. (2015). Maskless inverte pyramid texturizaion of silicon. Sci. Rep..

[21] Mikhlin Y.L., Vishnyakova E.A., Romanchenko A.S., Saikova S.V., Likhatski M.N., Larichev Y.V., Tuzikov F.V., Zaikovskii V.I., Zharkov S.M. (2014). Oxidation of Ag nanoparticles in aqueous media: Effect of particle size and capping. Appl. Surf. Sci..

[22] Aminuzzan M., Watanabe A., Miyashita T. (2010). Direct writing of conductive silver micropatterns on flexible polyimide film by laser-induced pyrolysis of silver nanoparticle-dispersed film. J. Nnaopart. Res..

[23] Lin Y., Zhang X., Bai S., Hu A. (2015). Photo-reduction of metallic ions doped in patterned polymer films for the fabrication of plasmonic photonic crystals. J. Mater. Chem. C.

[24] Peng K., Huang Z., Zhu J. (2004). Fabrication of Large-Area Silicon Nanowire p-n Jjunction Diode Arrays. Adv. Mater..

[25] Lajvardi M., Eshghi H., Izadifard M., Ghazi M.E., Goodarzi A. (2016). Effects of silver and gold catalytic activities on the structural and optical properties of silicon nanowires. Phys. E.

[26] Hutagalung S.D., Fadhali M.M., Areshi R.A., Tan F.D. (2017). Optical and Electrical Characteristics of Silicon Nanowires Prepared by Electroless Etching. Nanoscale Res. Lett..

[27] Peng H., Yang A.S., Xiong J.H. (2013). Green, microwave-assisted synthesis of silver nanoparticles using bamboo hemicelluloses and glucose in an aqueous medium. Carbohydr. Polym..

[28] Ameen S., Park D.R., Shin H.S. (2016). Silicon nanowires arrays for visible light driven photocatalytic degradation of rose bengal dye. J. Mater. Sci.-Mater. Electron..

[29] Le Ru E.C., Blackie E., Meyer M., Etchegoin P.G. (2007). Surface enhanced Raman scattering enhancement factors: A comprehensive study. J. Phys. Chem. C.

[30] Tang H., Meng G., Huang Q., Zhang Z., Huang Z., Zhu C. (2012). Arrays of cone-shaped ZnO nanorods decorated with Ag nanoparticles as 3D surface-enhanced Raman scattering substrates for rapid detection of trace polychlorinated biphenyls. Adv. Funct. Mater..

[31] Fang Y., Li X., Blinn K., Mahmoud M.A., Liu M. (2012). Resonant surface enhancement of Raman scattering of Ag nanoparticles on silicon substrates fabricated by dc sputtering. J. Vaccum Sci. Technol. A.

[32] Tian C.F., You H.J., Fang J.X. (2014). Three-dimensional noble-metal nanostructure: A new kind of substrate for sensitive, uniform, and reproducible surface-enhanced Raman scattering. Chin. Phys. B.

[33] Dong J., Qu S., Zheng H., Zhang Z., Li J., Huo Y., Li G. (2014). Simultaneous SEF and SERRS from silver fractal-like nanostructure. Sens. Actuators B-Chem..

[34] Lu J.F., Xu C.X., Nan H.Y., Zhu Q.X., Qin F.F., Manohari A.G., Wei M., Zhu Z., Shi Z.L., Ni Z.H. (2016). SERS-active ZnO/Ag hybrid WGM microcavity for ultrasensitive dopamine detection. Appl. Phys. Lett..

[35] Yu X.T., He X.X., Yang T.Q., Zhao L.T., Chen Q.C., Zhang S.J., Chen J.Q., Xu J.H. (2018). Sensitive determination of dopamine levels via surface-enhanced Raman scattering of Ag nanoparticle dimers. Int. J. Nanomed..

[36] Yu J.J., Shen M.Z., Liu S.Y., Li F., Sun D.P., Wang T.H. (2017). A simple technique for direct growth of Au into a nanoporous alumina layer on conductive glass as a reusable SERS substrate. Appl. Surf. Sci..

[37] Kang Y., Wu T., Han X.Z., Gu H.X., Zhang X. (2018). A needle-like reusable surface-enhanced Raman scattering substrate, and its application to the determination of acetamiprid by combining SERS and thin-layer chromatography. Microchim. Acta.

[38] Ghosh R., Ghosh J., Das R., Mawlong L.P.L., Paul K.K., Giri P.K. (2018). Multifunctional Ag nanoparticle decorated Si nanowires for sensing, photocatalysis and light emission applications. J. Colloid Interface Sci..

[39] Yang C.T., Wang J.L., Mei L.R., Wang X.Y. (2014). Enhanced Photocatalytic Degradation of Rhodamine B by Cu_2_O Coated Silicon Nanowire Arrays in Presence of H_2_O_2_. J. Mater. Sci. Technol..

[40] Singh A.N., Devnani H., Jha S., Ingole P.P. (2018). Fermi level equilibration of Ag and Au plasmonic metal nanoparticles supported on graphene oxide. Phys. Chem. Chem. Phys..

