# Supplementary material for: Reusable Surface-Enhanced Raman Spectroscopy Substrates Made of Silicon Nanowire Array Coated with Silver Nanoparticles Fabricated by Metal-Assisted Chemical Etching and Photonic Reduction
Source: Nanomaterials (Basel). 2019 Oct 28;9(11):1531. doi: 10.3390/nano9111531 (PMC6915563; doi:10.3390/nano9111531)
Supplement: Supplementary file 1 [file nanomaterials-09-01531-s001.pdf]

nanomaterials

Reusable Surface Enhanced Raman Spectroscopy Substrates Made of Silicon Nanowire Array Coated with Silver Nanoparticles Fabricated by Metal Assisted Chemical Etching and Photonic Reduction

Shi Bai[1,2], Yongjun Du[1], Chunyan Wang[3], Jian Wu[1*] and Koji Sugioka[2*]

[1] Institute of Laser Engineering, Beijing University of Technology, 100 Pingle Yuan, Beijing 100124, China
[2] Advanced Laser Processing Research Team, RIKEN Center for Advanced Photonics, RIKEN, 2–1 Hirosawa, Wako, Saitama 351–0198, Japan;
[3] State Key Laboratory of Space Medicine Fundamentals and Application, Chinese Astronaut Science Researching and Training Center, Beijing, 100094, China;
* Corresponding author: 5777@bjut.edu.cn (J.W.); ksugioka@riken.jp (K.S.);

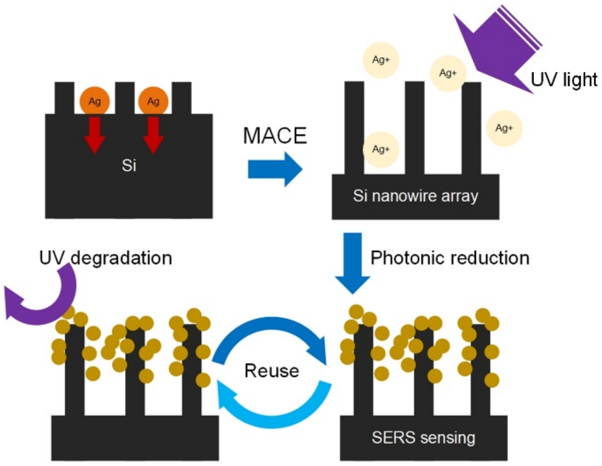

Figure S1. Schematic diagram of fabrication of silicon nanowire array coated with silver nanoparticles SERS substrate with high sensitivity and reusability by MACE and successive photonic reduction.

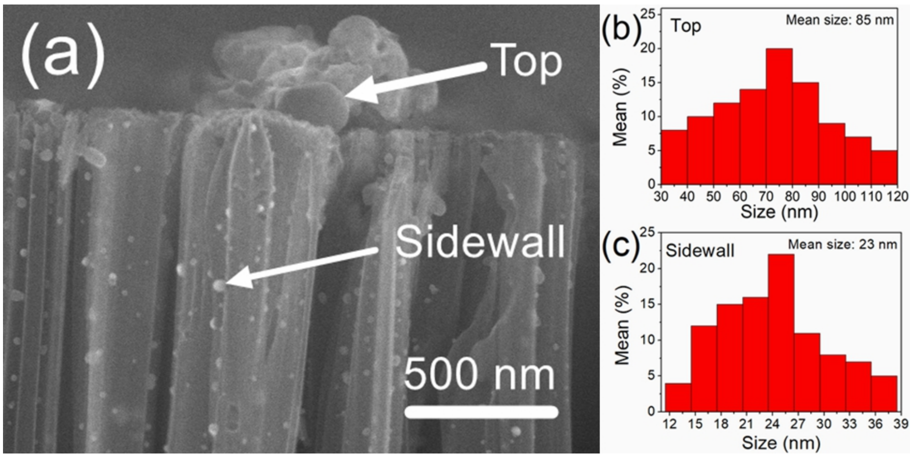

Figure S2. (a) SEM image of silicon nanowire array coated with silver nanoparticles by UV irradiation for 10 min. (b) and (c) The size distribution of Ag nanoparticle on the top surface and sidewall of nanowire array.

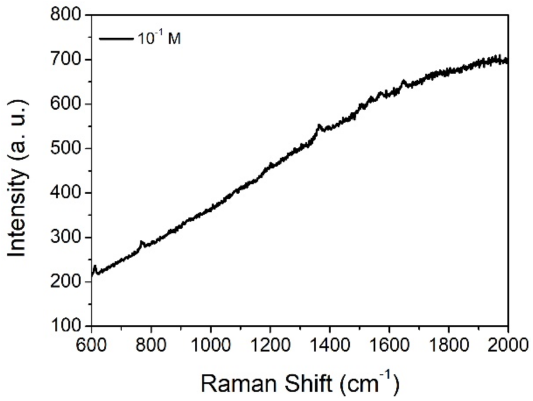

Figure S3. The Raman spectrum of R6G on silicon wafer. The concentration of R6G is 10^{-1}M.

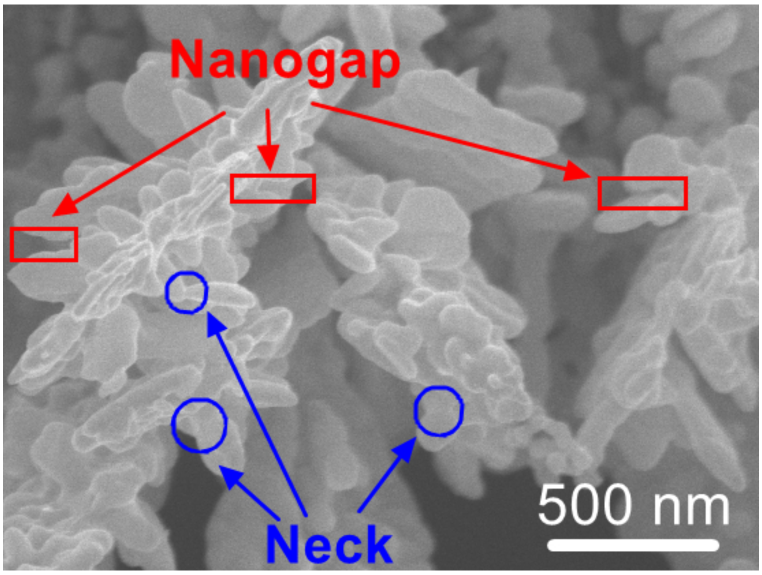

Figure S4. SEM image of branched silver dendrites. The nanogaps and neck areas on branched silver dendrites are marked by red rectangles and blue circles, respectively.

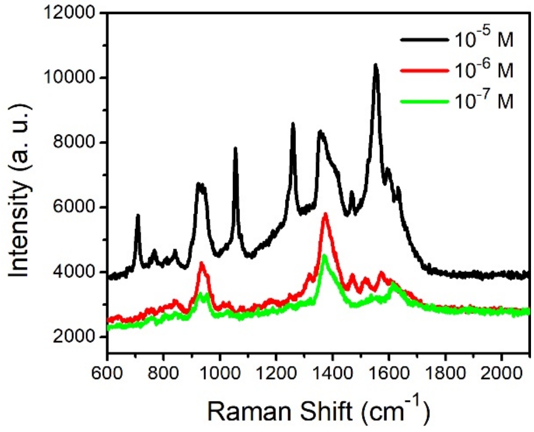

Figure S5. SERS spectra of DA solutions with different concentrations on silicon nanowires coated with silver nanoparticles. The laser power for DA Raman measurement is 0.05 mW.

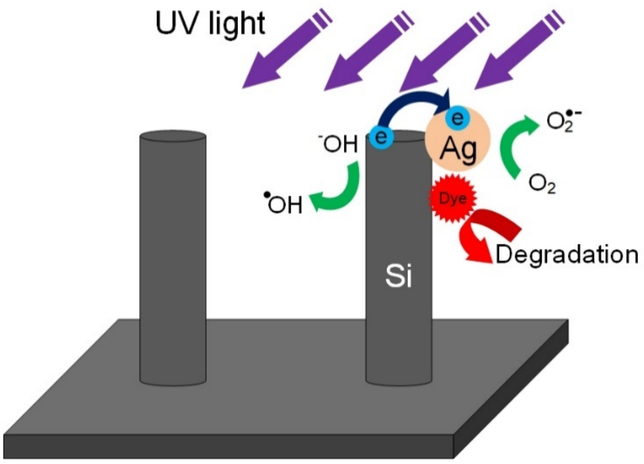

Figure S6. Schematic diagram of photocatalytic degradation of dye molecule on Ag NP/Si NW substrate by UV light irradiation.

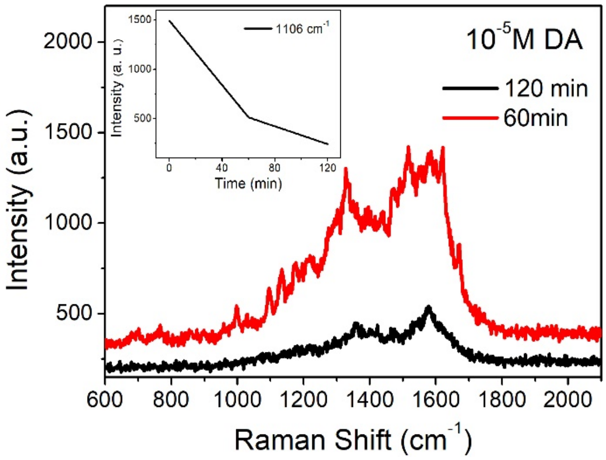

Figure S7. The Raman spectrum of DA on Ag-NP/Si-NW array SERS substrate after 60 min and 120 min UV photodegradation. The concentration of DA is 10^{-5}M. Insert: The variation of Raman peak intensity at 1106 cm^{-1} treated by UV photodegradation.

© 2019 by the authors. Submitted for possible open access publication under the terms and conditions of the Creative Commons Attribution (CC BY) license (http://creativecommons.org/licenses/by/4.0/).
